# Association of cardiac autonomic dysfunction with higher levels of plasma lipid metabolites in recent-onset type 2 diabetes

**DOI:** 10.1007/s00125-020-05310-5

**Published:** 2020-10-21

**Authors:** Dan Ziegler, Alexander Strom, Klaus Straßburger, Birgit Knebel, Gidon J. Bönhof, Jörg Kotzka, Julia Szendroedi, Michael Roden, for the German Diabetes Study group

**Affiliations:** 1 Institute for Clinical Diabetology, German Diabetes Center (DDZ), Leibniz Center for Diabetes Research at Heinrich Heine University, Düsseldorf, Germany; 2 Division of Endocrinology and Diabetology, Medical Faculty, Heinrich Heine University, Düsseldorf, Germany; 3 German Center for Diabetes Research (DZD), München-Neuherberg, Germany; 4 Institute for Biometrics and Epidemiology, German Diabetes Center (DDZ), Leibniz Center for Diabetes Research at Heinrich Heine University, Düsseldorf, Germany; 5 Institute for Clinical Biochemistry and Pathobiochemistry, German Diabetes Center (DDZ), Leibniz Center for Diabetes Research at Heinrich Heine University, Düsseldorf, Germany

**Keywords:** Cardiac autonomic dysfunction, Heart rate variability, Lipid metabolites, Recent-onset type 2 diabetes

## Abstract

**Aims/hypothesis:**

Emerging evidence suggests that in addition to hyperglycaemia, dyslipidaemia could represent a contributing pathogenetic factor to diabetic neuropathy, while obesity and insulin resistance play a role in the development of diabetic cardiac autonomic neuropathy (CAN) characterised by reduced heart rate variability (HRV), particularly in type 2 diabetes. We hypothesised that distinct lipid metabolites are associated with diminished HRV in recent-onset type 2 diabetes rather than type 1 diabetes.

**Methods:**

We analysed 127 plasma lipid metabolites (11 acylcarnitines, 39 NEFA, 12 sphingomyelins (SMs), 56 phosphatidylcholines and nine lysophosphatidylcholines) using MS in participants from the German Diabetes Study baseline cohort recently diagnosed with type 1 (*n* = 100) and type 2 diabetes (*n* = 206). Four time-domain HRV indices (number of normal-to-normal (NN) intervals >50 ms divided by the number of all NN intervals [pNN50]; root mean square of successive differences [RMSSD]; SD of NN intervals [SDNN]; and SD of differences between adjacent NN intervals) and three frequency-domain HRV indices (very-low-frequency [VLF], low-frequency [LF] and high-frequency [HF] power spectrum) were computed from NN intervals recorded during a 3 h hyperinsulinaemic–euglycaemic clamp at baseline and in subsets of participants with type 1 (*n* = 60) and type 2 diabetes (*n* = 95) after 5 years.

**Results:**

In participants with type 2 diabetes, after Bonferroni correction and rigorous adjustment, SDNN was inversely associated with higher levels of diacyl-phosphatidylcholine (PCaa) C32:0, PCaa C34:1, acyl-alkyl-phosphatidylcholine (PCae) C36:0, SM C16:0 and SM C16:1. SD of differences between NN intervals was inversely associated with PCaa C32:0, PCaa C34:1, PCaa C34:2, PCae C36:0 and SM C16:1, and RMSSD with PCae C36:0. For VLF power, inverse associations were found with PCaa C30:0, PCaa C32:0, PCaa C32:1, PCaa C34:2 and SM C16:1, and for LF power inverse associations were found with PCaa C32:0 and SM C16:1 (*r* = −0.242 to *r* = −0.349; *p* ≤ 0.0005 for all correlations). In contrast, no associations of lipid metabolites with measures of cardiac autonomic function were noted in participants recently diagnosed with type 1 diabetes. After 5 years, HRV declined due to ageing rather than diabetes, whereby prediction analyses for lipid metabolites were hampered.

**Conclusions/interpretation:**

Higher plasma levels of specific lipid metabolites are closely linked to cardiac autonomic dysfunction in recent-onset type 2 diabetes but not type 1 diabetes, suggesting a role for perturbed lipid metabolism in the early development of CAN in type 2 diabetes.

**Figure:**
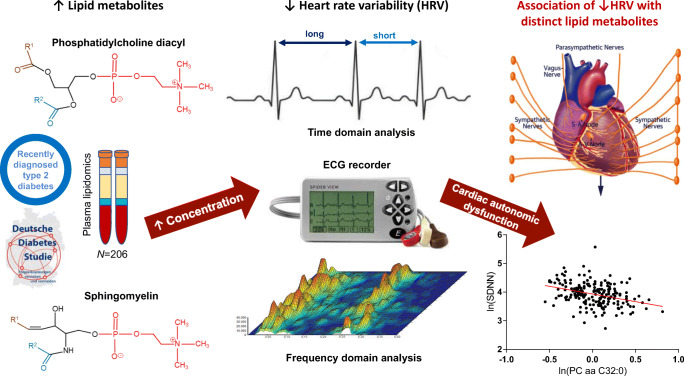
Graphical abstract

**Electronic supplementary material:**

The online version of this article (10.1007/s00125-020-05310-5) contains peer-reviewed but unedited supplementary material, which is available to authorised users.

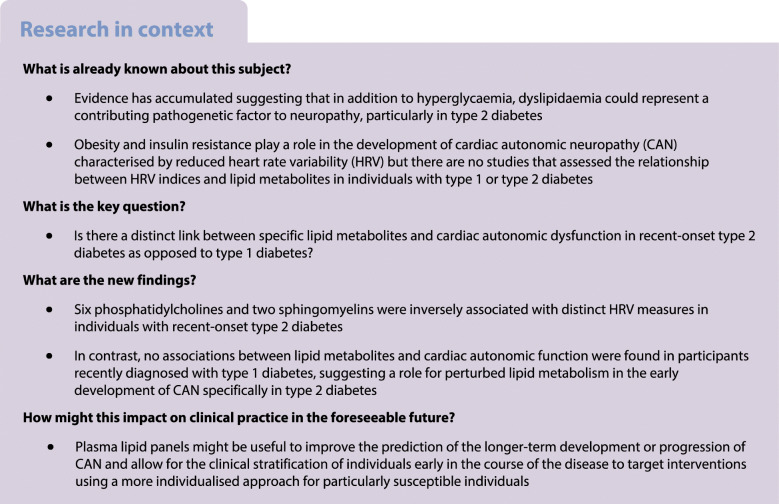



## Introduction

Cardiovascular autonomic neuropathy (CAN), with its hallmark reduced heart rate variability (HRV), affects approximately 20% of people with diabetes and predicts an increased risk of major cardiac events and mortality [1]. We recently demonstrated that lower HRV, indicating diminished cardiovagal tone, in recent-onset type 2 diabetes is associated with insulin resistance, hepatic steatosis and blunted cardiorespiratory fitness, suggesting that these components could play an important role in the early development of CAN, apart from well-known risk factors such as higher age, obesity, hypertension or poor glycaemic control [2]. Indeed, it has been emphasised that hyperglycaemia cannot be considered as the sole factor responsible for the development of diabetic complications, particularly in patients with type 2 diabetes. Instead, an interplay of the aforementioned factors that have an impact on the adipose tissue fatty acid metabolism could underlie the onset and progression of diabetic microvascular complications including neuropathy [3].

There is accumulating evidence suggesting that in addition to hyperglycaemia, dyslipidaemia could represent a contributing pathogenetic factor to neuropathy, particularly for type 2 diabetes. Since both hyperglycaemia and dyslipidaemia affect multiple cells in the peripheral nervous system, including neuronal axons, Schwann cells and dorsal root ganglia, deciphering the mechanisms by which perturbed glucose and lipid metabolism converge to result in nerve damage could foster the development of novel lipid-based disease-modifying treatments for diabetic neuropathy [4]. Previous studies assessing large cohorts of individuals with type 2 diabetes point to a link between plasma lipid levels and diabetic neuropathies. The Fremantle Diabetes Study reported that the use of fibrates and statins was associated with lower risks of developing polyneuropathy over 5 years [5]. Furthermore, the Anglo-Danish-Dutch Study of Intensive Treatment in People With Screen-Detected Diabetes in Primary Care (ADDITION)-Denmark study showed that hypertriacylglycerolaemia was associated with prevalent CAN [6], while low HDL-cholesterol levels were predictors of incident polyneuropathy over 13 years [7].

The past decade has witnessed novel technologies such as metabolomics aimed at the extensive characterisation and quantification of global metabolites from both endogenous and exogenous sources in the context of insulin resistance, the metabolic syndrome and type 2 diabetes [8–11]. As a subfield of metabolomics, lipidomics emerged to investigate the relationship of dysregulation in lipid metabolism and pathogenesis of type 2 diabetes [12] and diabetic neuropathy [13].

To date, there are no studies that assess the relationship between cardiac autonomic function and metabolomic profiles including lipidomics in individuals with type 1 or type 2 diabetes. Using targeted fasting plasma metabolomic analysis, we previously reported both similarities and diabetes type-specific differences in the metabolite patterns when comparing participants with type 1 diabetes and type 2 diabetes from the German Diabetes Study (GDS) baseline cohort [14]. Given the complex interplay between hyperglycaemia, insulin resistance, obesity and lipid metabolism as the putative factors contributing to diabetic neuropathy, we hypothesised that a distinct link exists between specific lipid metabolites and cardiac autonomic dysfunction in recent-onset type 2 diabetes as opposed to type 1 diabetes.

## Methods

### Study participants

Individuals recently diagnosed with diabetes (known diabetes duration ≤1 year) and glucose-tolerant control individuals were recruited consecutively from the baseline cohort of the GDS. The GDS is a prospective observational study investigating the natural course of metabolic alterations and the development of chronic diabetic complications (ClinicalTrial.gov registration no: NCT01055093). The study was approved by the local ethics committee of Heinrich Heine University, Düsseldorf, Germany, and informed written consent was obtained from all participants prior to participation. The study design and cohort profile of the GDS have been described in detail previously [15]. The present cross-sectional analysis of lipid metabolites and HRV measures included 100 consecutive participants with type 1 diabetes and 206 consecutive participants with type 2 diabetes. The prospective analysis of HRV indices included 60 individuals with type 1 diabetes and 95 individuals with type 2 diabetes who reached the 5 year follow-up.

### Hyperinsulinaemic–euglycaemic clamp

All participants underwent an IVGTT followed by a modified Botnia clamp test with [6,6-^2^H_2_]glucose to measure whole-body insulin sensitivity as previously described [15]. Whole-body insulin sensitivity (*M* value; [μmol glucose] [body weight in kg]^−1^ min^−1^) was calculated from the difference between mean glucose infusion rates during steady state in the last 30 min of the clamp with glucose space correction [15].

### HRV

R–R intervals were measured in the supine position during a hyperinsulinaemic–euglycaemic clamp over 3 h using a digital Spider View Holter recorder with seven electrodes to record three-channel ECGs (Sorin Group, Munich, Germany) as previously described [16]. Time-domain HRV measures included the SD of differences between adjacent normal-to-normal (NN) intervals, SD of all NN intervals (SDNN), the number of pairs of adjacent NN intervals differing by >50 ms in the entire recording divided by the total number of NN intervals (pNN50), and the root mean square of successive differences (RMSSD). Frequency-domain HRV indices included the very-low-frequency (VLF) band (0.003–0.04 Hz), low-frequency (LF) band (0.04–0.15 Hz) and high-frequency (HF) band (0.15–0.4 Hz) [17, 18].

Cardiovascular autonomic function tests were performed to diagnose CAN, including seven HRV indices measured during spontaneous breathing over 5 min (coefficient of R–R interval variation, VLF and LF power), at deep breathing (expiration-to-inspiration [E/I] ratio), after standing up (max/min 30:15 ratio) and in response to a Valsalva manoeuvre (Valsalva ratio) using VariaCardio TF5 (MIE Medical Research, Leeds, UK), as previously described [19]. Age-dependent lower limits of normal were defined at the fifth percentile of healthy participants. The systolic BP response to standing up was measured over 3 min using −27 mmHg as an age-independent lower limit of normal. Borderline CAN was assumed if two out of seven indices were abnormal, while definite CAN was diagnosed if three or more out of seven indices were abnormal [19]. Brief explanations for the physiological basis of the various HRV indices are included into the electronic supplementary material [ESM] Methods.

### Lipid metabolites

Fasting sodium heparinate plasma samples were rapidly frozen and stored at −80°C. Targeted metabolic profiling of 127 blood lipid metabolites (11 acylcarnitines, 39 NEFA, 12 sphingomyelins (SMs), 56 phosphatidylcholines and nine lysophosphatidylcholines) was performed with the X MetaDis/DQTM Kit at Biocrates Life Sciences (Innsbruck, Austria) as previously described [14]. In brief, this targeted IDQ metabolomics platform enables the investigation of various metabolites using GC–, LC–, flow injections analysis–MS and mass spectrometric procedures for more specialised identifications. The limit of detection (LOD) was determined for each metabolite from the signal-to-noise ratio. Metabolites were included in further analyses if values exceeded the respective LOD and could be detected in >95% of the samples examined. Preanalytical stability of metabolites was tested during three different handling procedures with replicate samples (*n* = 10 each). Metabolites were excluded due to insufficient reliability (CV >30%). This cut-off value is approximately three times the maximum variation per metabolite observed in independent comparative metabolome studies using the same technology [20].

### Statistical analysis

Data are presented as mean ± SD, median (first quartile, third quartile) or percentages. Categorical variables were compared using χ^2^ test and expressed as percentages of participants. Continuous data were assessed using the parametric *t* test or non-parametric Mann–Whitney *U* test. Metabolite concentrations were log transformed (log_*e*_) because of their skewed distribution and adjusted for plate effects. Correlations between two variables were determined using Spearman rank correlation. For multiple linear regression analyses, dependent variables with skewed distribution were log transformed (log_*e*_) before analyses. The analyses were adjusted for age, sex, BMI, smoking and antihypertensive and lipid-lowering drugs. All statistical tests were two-sided and the level of significance was set at α = 0.05. *p* values obtained from univariate correlation analyses were adjusted for multiple comparisons using Bonferroni correction considering seven HRV indices (pNN50, RMSSD, SDNN, SD of differences between adjacent NN intervals, and VLF, LF and HF bands) and the number of metabolites in the corresponding lipid class (11 acylcarnitines, 39 NEFA, 12 SMs, 56 phosphatidylcholines and nine lysophosphatidylcholines).

To consider the physiological decline in HRV over the 5-year follow-up period, age-dependent regressions of the corresponding HRV variables were calculated in 167 glucose-tolerant individuals from the GDS study (mean ± SD: age 45.5 ± 14.1 years; BMI 26.9 ± 4.8 kg/m^2^; HbA_1c_ 33.2 ± 3.2 mmol/mol [5.2 ± 0.3%]), 108 (65%) of whom were male. Using the resulting equations, we determined the magnitude of the physiological HRV decline over the 5 years for each HRV measure and added these values to the corresponding individual HRV values for participants with type 1 and type 2 diabetes (ESM Table 7). Wilcoxon signed-rank test was used to analyse the changes in HRV indices from baseline to 5 years before and after adjustment for the 5 year follow-up period. All analyses were performed using SPSS version 22.0 software (IBM Corporation, Chicago, IL, USA).

## Results

### Cross-sectional analysis

The demographic, clinical and HRV data for the participants with type 1 and type 2 diabetes are listed in Table 1. Compared with type 1 diabetes individuals, those with type 2 diabetes were older, had higher BMI, lower *M* value and were more frequently taking glucose-lowering, lipid-lowering, and antihypertensive drugs (all *p* < 0.05). No differences between the groups were found for the remaining demographic and clinical variables after adjustment for sex, age, BMI and smoking status. Prior to adjustment, all seven HRV indices were lower in participants with type 2 diabetes than in those with type 1 diabetes but after adjustment for sex, age, BMI, smoking status, HbA_1c_, fasting blood glucose, *M* value, triacylglycerols, cholesterol, HDL-cholesterol, LDL-cholesterol, creatinine, proteinuria, insulin therapy, oral glucose-lowering drugs, antihypertensive drugs and lipid-lowering drugs, no differences in any of the HRV measures were found between the groups (Table 1).

**Table 1:** Demographic and clinical characteristics at baseline

Variable	Type 1 diabetes	Type 2 diabetes	*p* value^a^
*n* (% male)	100 (64)	206 (67)	0.609
Age, years	34.5 ± 13.0	53.5 ± 10.9	<0.0001
BMI, kg/m^2^	24.6 ± 4.2	31.8 ± 6.0	<0.0001
Current smoking status, % yes	24.0	21.4	0.661
Heart rate, beats/min^b^	69.4 ± 10.6	69.9 ± 10.2	0.445
Systolic BP, mmHg^b^	117 ± 12	132 ± 15	<0.0001
Diastolic BP, mmHg^b^	65.8 ± 8.5	73.6 ± 8.9	<0.0001
Triacylglycerols, mmol/l^b^	0.76 (0.56, 1.13)	1.48 (1.04, 2.20)	<0.0001
Cholesterol, mmol/l^b^	4.65 ± 0.98	5.38 ± 1.13	<0.0001
HDL-cholesterol, mmol/l^b^	1.52 ± 0.45	1.21 ± 0.33	<0.0001
LDL-cholesterol, mmol/l^b^	2.69 ± 0.89	3.39 ± 0.98	<0.0001
Creatinine, nmol/l	80.2 ± 13.7	80.3 ± 15.3	0.928
HbA_1c_, mol/mmol	50.5 ± 14.8	47.2 ± 9.9	0.080
HbA_1c_, %	6.8 ± 1.4	6.5 ± 0.9	0.080
Fasting glucose, mmol/l	7.87 ± 3.00	7.16 ± 1.61	0.428
*M* value, μmol kg^−1^ min^−1 b^	43.8 (32.2, 56.6)	33.9 (23.3, 42.7)	<0.0001*
Diabetes duration, days	204 ± 95	195 ± 88	0.405
Insulin treatment, %	89.0	7.8	<0.0001
Glucose-lowering drugs, %	16.0	55.8	<0.0001
Antihypertensive drugs, %	9.0	58.3	<0.0001
Lipid-lowering drugs (total/statins), %	1.0 (1.0)	22.8 (19.4)	<0.0001
Albuminuria, mg/l^b^	14.8 ± 9.8	22.1 ± 43.8	0.038
Subclinical/borderline CAN, %	3.4	5.0	0.756
Definite CAN, %	2.2	3.3	1.000
Time-domain HRV indices^c^			
pNN50, %	12.4 (5.6, 25.2)	4.9 (1.4, 14.5)	<0.0001
RMSSD, ms	37.2 (27.1, 50.8)	27.0 (19.9, 41.3)	<0.0001
SDNN, ms	66.0 (51.2, 87.1)	50.2 (39.1, 64.4)	<0.0001
SD of differences between adjacent NN intervals, ms	78.5 (59.6, 94.5)	59.8 (46.2, 74.5)	<0.0001
Frequency-domain HRV indices^c^			
VLF power, ms^2^	2078 (1314, 3696)	1437 (846, 2381)	<0.0001
LF power, ms^2^	1310 (753, 2115)	586 (329, 1057)	<0.0001
HF power, ms^2^	402 (207, 701)	168 (88, 338)	<0.0001

Data are presented as %, mean±SD, or median (first quartile, third quartile)

^a^*p* value prior to adjustment

^b^Group comparison adjusted for sex, age, BMI and smoking status; *p<0.05 vs type 1 diabetes after adjustment for sex, age, BMI and smoking status

^c^Group comparison adjusted for sex, age, BMI, smoking status, HbA_1c_, fasting blood glucose, *M* value, triacylglycerols, cholesterol, HDL-cholesterol, LDL-cholesterol, creatinine, proteinuria, insulin therapy, oral glucose-lowering drugs, antihypertensive drugs and lipid-lowering drugs. There were no differences between the groups for the HRV indices after adjustment

hsCRP, high-sensitivity C-reactive protein

**Fig. 1:**
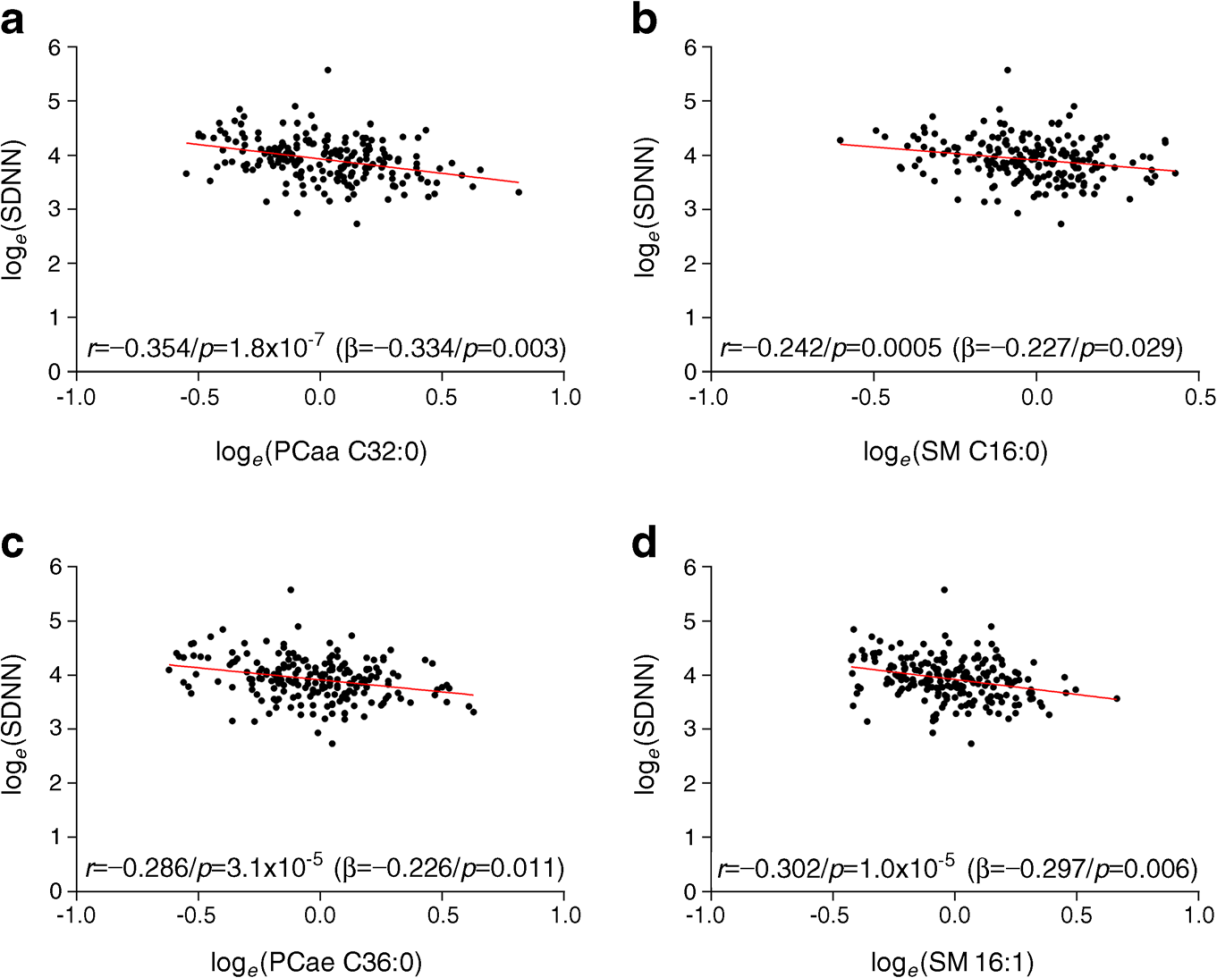
(**a**–**d**) Representative examples for inverse correlations of lipid metabolites with SDNN in participants recently diagnosed with type 2 diabetes

The associations of higher levels of lipid metabolites with lower HRV indices in participants with recent-onset type 2 diabetes are shown in Table 2. After Bonferroni correction and following adjustment for sex, age, BMI, smoking status, HbA_1c_, fasting blood glucose, *M* value, triacylglycerols, cholesterol, HDL-cholesterol, LDL-cholesterol, creatinine, proteinuria, insulin therapy, oral glucose-lowering drugs, antihypertensive drugs and lipid-lowering drugs, the following associations were noted among the time-domain HRV indices: SDNN was inversely associated with higher levels of diacyl-phosphatidylcholine (PCaa) C32:0 and PCaa C34:1, and acyl-alkyl-phosphatidylcholine (PCae) C36:0, as well as SM C16:0 and SM C16:1; SD for differences between adjacent NN intervals was inversely associated with PCaa C32:0, PCaa C34:1, PCaa C34:2 and PCae C36:0, as well as SM C16:1 and RMSSD was inversely associated with PCae C36:0 (all *p* < 0.05). Following Bonferroni correction and the aforementioned adjustments, the following inverse associations were noted among the frequency-domain HRV indices: VLF power was associated with PCaa C30:0, PCaa C32:0, PCaa C32:1 and PCaa C34:2, as well as SM C16:1; and LF power was associated with PCaa C32:0 and SM C16:1 (all *p* < 0.05). The remaining relationships were either not statistically significant or lost statistical significance after Bonferroni correction or following adjustment. Representative examples for the inverse correlations of lipid metabolites with SDNN in participants recently diagnosed with type 2 diabetes are shown in Fig. 1a–d.

**Table 2:** Inverse associations of lipid metabolites with HRV measures in participants with recent-onset type 2 diabetes

Metabolite	Time domain HRV indices								Frequency domain HRV indices					
	pNN50		RMSSD		SDNN		SD		VLF		LF		HF	
	*r*	*p*	*r*	*p*	*r*	*p*	*r*	*p*	*r*	*p*	*r*	*p*	*r*	*p*
PCaa C30:0	−0.225	0.001	−0.217	0.002	−0.285	<0.0001*	−0.266	0.0001*	−0.289	0.0001*^†^	−0.244	0.001	−0.185	0.009
PCaa C32:0	−0.263	0.0001*	−0.260	0.0002^†^	−0.345	<0.0001*^†^	−0.340	<0.0001*^†^	−0.349	<0.0001*^†^	−0.295	<0.0001*^†^	−0.249	0.0004^†^
PCaa C32:1	−0.189	0.007	−0.166	0.017	−0.260	0.0002^†^	−0.258	0.0002	−0.268	0.0001*^†^	−0.192	0.006	−0.109	0.124
PCaa C34:1	−0.213	0.002^†^	−0.197	0.005^†^	−0.266	0.0001*^†^	−0.265	0.0001*^†^	−0.259	0.0002^†^	−0.202	0.004^†^	−0.148	0.037
PCaa C34:2	−0.198	0.005^†^	−0.183	0.009^†^	−0.259	0.0002^†^	−0.265	0.0001*^†^	−0.275	<0.0001*^†^	−0.218	0.002^†^	−0.168	0.018^†^
PCae C36:0	−0.256	0.0002^†^	−0.263	0.0001*^†^	−0.286	<0.0001*^†^	−0.271	<0.0001*^†^	−0.237	0.001^†^	−0.254	0.0003^†^	−0.260	0.0002^†^
SM C16:0	−0.208	0.003^†^	−0.174	0.012^†^	−0.242	0.0005*^†^	−0.233	0.001^†^	−0.201	0.004^†^	−0.203	0.004^†^	−0.173	0.015^†^
SM C16:1	−0.197	0.015^†^	−0.169	0.015^†^	−0.302	<0.0001*^†^	−0.292	<0.0001*^†^	−0.254	0.0003*^†^	−0.260	0.0002*^†^	−0.140	0.049^†^

Metabolite nomenclature indicates C*x*:*y,* whereby *x* is the number of carbons in the fatty acid side chain, *y* is the number of double bonds in the fatty acid side chain

*p<0.05 after Bonferroni correction for multiple testing (each group of metabolites was tested separately)

^†^*p*<0.05 after adjustment for sex, age, BMI, smoking status, HbA_1c_, fasting blood glucose, *M* value, triacylglycerols, cholesterol, HDL-cholesterol, LDL-cholesterol, creatinine, proteinuria, insulin therapy, oral glucose-lowering drugs, antihypertensive drugs and lipid-lowering drugs

In the participants with recent-onset type 2 diabetes, multiple associations were also observed between the seven HRV indices and multiple other lipid metabolites from the five classes investigated. However, after Bonferroni correction or after adjustment, these associations lost statistical significance. The relationships between HRV measures and NEFA, phosphatidylcholines and lysophosphatidylcholines, SMs and acylcarnitines are shown in ESM Table 1, ESM Table 2, ESM Table 3 and ESM Table 4, respectively.

No associations between lipid metabolites and HRV indices were found either after Bonferroni correction or after adjustment in the group with recent-onset type 1 diabetes. The relationship between lipid metabolites and HRV measures in participants with recent-onset type 1 diabetes is shown in ESM Table 5 (only bivariate correlations with *p* < 0.005 for at least one HRV index before adjustment are listed). After adjustment for sex, age, BMI, smoking status, HbA_1c_, fasting blood glucose, *M* value, triacylglycerols, cholesterol, HDL-cholesterol, LDL-cholesterol, creatinine, proteinuria, insulin therapy, oral glucose-lowering drugs, antihypertensive drugs and lipid-lowering drugs, statistical significance was lost (*p* > 0.05 for all correlations).

### Prospective analysis

The demographic and clinical characteristics of the subsets of participants who completed the 5 year HRV follow-up are shown in ESM Table 6. In both diabetes groups, triacylglycerols, HDL-cholesterol, LDL-cholesterol and HbA_1c_ increased, while albuminuria decreased from baseline to 5 years (all *p* < 0.05). In participants with type 1 diabetes, BMI and total cholesterol increased and *M* value decreased, while in those with type 2 diabetes, fasting glucose and the percentage of antihypertensive drug users increased and creatinine decreased from baseline to 5 years (all *p* < 0.05).

Table 3 shows the HRV indices in subsets of individuals who completed the 5 year HRV follow-up. In unadjusted analyses in both diabetes groups LF and HF power decreased, while in addition in participants with type 1 diabetes pNN50 decreased (all *p* < 0.05). After adjustment for the 5 year follow-up period, SD for differences between adjacent NN intervals increased in both diabetes groups, while VLF power increased only in the group with type 1 diabetes (all *p* < 0.05). The sum of the original values and the added age-dependent 5 year physiological changes in the HRV indices at 5 years are shown in ESM Table 7 together with the corresponding equations and syntaxes obtained from the regressions computed in 167 glucose-tolerant control individuals from the GDS study. There were no associations between the baseline levels of lipid metabolites and the changes in HRV indices over 5 years (data not shown).

**Table 3:** Follow-up of HRV indices after 5 years

HRV variable	Type 1 diabetes (*n*=60)		Type 2 diabetes (*n*=95)	
	Baseline	5 years	Baseline	5 years
Time-domain HRV indices				
pNN50, %	12.6 (6.4, 25.5)	7.8 (2.5, 23.6)*	5.3 (2.0, 18.3)	4.5 (1.3, 12.1)
RMSSD, ms	37.8 (28.7, 52.0)	31.0 (23.1, 50.5)	28.8 (21.4, 43.4)	27.3 (20.2, 39.1)
SDNN, ms	66.7 (52.5, 86.7)	66.0 (47.0, 85.6)	51.9 (41.1, 67.5)	48.9 (39.3, 66.4)
SD of differences between adjacent NN intervals, ms	72.8 (58.9, 90.5)	76.5 (61.2, 93.6)^†^	62.8 (48.7, 79.9)	65.1 (51.7, 83.2)^†^
Frequency-domain HRV indices				
VLF power, ms^2^	2015 (1437, 3263)	2377 (1501, 3428)^†^	1466 (871, 2601)	1421 (830, 2408)
LF power, ms^2^	1209 (748, 2143)	1118 (542, 1813)*	659 (391, 1170)	503 (249, 940)*
HF power, ms^2^	351 (188, 743)	251 (119, 681)*	170 (98, 354)	146 (80, 352)*

Data are shown as median (first, third quartile)

*p<0.05 vs baseline before adjustment; ^†^*p*<0.05 vs baseline after adjustment for the 5 year follow-up period (Wilcoxon signed-rank test)

## Discussion

The results of this study demonstrate that higher plasma levels of distinct lipid metabolites, namely phosphatidylcholines (five diacyl and one acyl-alkyl) and SMs (C16:0 and C16:1) are linked to cardiac autonomic dysfunction, particularly to lower cardiovagal tone, in individuals recently diagnosed with type 2 diabetes. In contrast, no associations of lipid metabolites with cardiac autonomic function were found in participants with recent-onset type 1 diabetes, suggesting a role for perturbed lipid metabolism in the early development of CAN specifically in type 2 diabetes. However, the 5 year follow-up period was too short to detect clinically relevant deterioration in HRV in excess of the physiological effect of ageing and thereby to allow for analyses of the predictive value of lipid metabolites in the development or progression of CAN.

There are no published studies available with which our findings could be directly compared. Previous cohort studies focused on the predictive value of various lipid metabolites, in particular phosphatidylcholine consumption, on incident type 2 diabetes as well as CVD and mortality risk. Phosphatidylcholines are a class of phospholipids that incorporate choline as a headgroup. They are a major component of biological membranes and can be easily obtained from a variety of readily available sources, such as egg yolk or soybeans, from which they are mechanically or chemically extracted using hexane. In the Nurses’ Health Study (NHS), NHS II and the Health Professionals Follow-up Study (HPFS), dietary intake of phosphatidylcholine was associated with an increased risk of incident type 2 diabetes [21]. Moreover, in the NHS and HPFS, higher phosphatidylcholine consumption was associated with increased all-cause and cardiovascular mortality risk, especially in patients with diabetes, independent of traditional risk factors [22]. Recent animal studies point to a mechanistic link between intestinal microbial metabolism of the choline moiety in dietary phosphatidylcholine and CVD through the production of a proatherosclerotic metabolite, trimethylamine-*N*-oxide (TMAO), which is associated with an increased risk of incident major adverse cardiovascular events [23]. However, the way in which TMAO could also play a role in the context of type 2 diabetes and CAN remains unknown.

The present study also shows associations between higher concentrations of SMs C16:0 and C16:1 and lower HRV indices, largely indicating diminished cardiovagal tone. Sphingolipids are complex lipids that are particularly abundant in nervous tissue and are implicated not only in a number of neurological diseases but also in insulin-resistant conditions such as diabetes or non-alcoholic steatohepatitis [24, 25]. Higher concentrations of specific SMs have recently been found to predict incident type 2 diabetes in prospective sphingolipidomics studies over 6 and 11 years, respectively [26, 27]. The formation of atypical neurotoxic deoxysphingolipids has been identified to play a causative role in the development of hereditary sensory and autonomic neuropathy type 1 (HSAN1) [12]. Of note, 1-deoxysphingolipids have also been found to be elevated in individuals with type 2 diabetes and in non-diabetic individuals with the metabolic syndrome [28]. Moreover, 1-deoxysphingolipid levels were also increased in individuals with type 2 diabetes who had polyneuropathy, when compared with healthy individuals, but there were no correlations between these levels and peripheral nerve function tests or the clinical neuropathy stages [12]. In an open-label clinical trial in individuals with primary hypercholesterolemia or mixed dyslipidaemia, treatment with fenofibrate for 6 weeks resulted in lowering of plasma 1-deoxysphingolipid levels [29]. An open-label trial in individuals with type 1 diabetes showed that supplementation with seal oil ω-3 polyunsaturated fatty acids over 12 years was associated with an increase in corneal nerve fibre length [30]. In the Fenofibrate Intervention and Event Lowering in Diabetes (FIELD) study, treatment with fenofibrate for 5 years in individuals with type 2 diabetes was associated with a lower risk of amputations in the lower limbs, particularly minor amputations in the absence of peripheral arterial disease, which were predicted by neuropathy [31]. Moreover, the de novo sphingolipid synthesis pathway is considered a promising target for pharmacological intervention in insulin resistance. It has been shown that inhibition of serine palmitoyltransferase, the first enzyme in the sphingolipid biosynthesis pathway, increases insulin sensitivity [32]. Thus, there is accumulating evidence supporting the notion that lipidomics-based novel disease-modifying treatment approaches could merit further investigation in type 2 diabetes patients with polyneuropathy and CAN.

Other lipid metabolites contributing to an increased risk of diabetes and CVD include acylcarnitines [33–35]. Carnitine is a quaternary ammonium compound involved in fatty acid metabolism, maintaining the balance between free and esterified CoA, which is crucial for normal cell function. In humans, carnitine exists as free active l-carnitine or as acylcarnitines (i.e. esterified forms with various chain lengths) [36]. Carnitine plays an important role in transporting long-chain fatty acids from the cytosol to the mitochondrial matrix, where β-oxidation takes place, and accumulation of acylcarnitines may reflect dysregulated fatty acid oxidation [37]. In a Chinese study, a panel of acylcarnitines mainly involved in mitochondrial lipid dysregulation predicted incident type 2 diabetes beyond conventional risk factors [33]. Among individuals with suspected stable angina pectoris, elevated serum even-chained acylcarnitines were associated with increased risk of cardiovascular death and to a lesser degree with acute myocardial infarction, again independent of traditional risk factors [35]. Although we observed inverse associations of four acylcarnitines with several HRV indices (see ESM Table 4), statistical significance was lost after Bonferroni correction, while none of the ten NEFAs showed associations after rigorous adjustment (see ESM Table 1). However, since a type II error cannot be excluded, it is conceivable that if these associations were true, they would obviously be weaker than for the aforementioned lipid metabolites.

The potential source of bias resulting from the disparity in the use of lipid-lowering medications, the vast majority of which were statins, in 23% and 1% of participants with type 2 and type 1 diabetes, respectively, deserves comment. To address this aspect, all relevant analyses were adjusted for lipid-lowering medication. In epidemiological studies, the statin-mediated lipidomic changes in individuals with the metabolic syndrome or type 2 diabetes showed a significant shift towards the lipid profile of control individuals, indicative of a marked trend towards a normolipidemic phenotype [38]. Moreover, administration of rosuvastatin for 3–8 weeks in individuals with hyperlipidaemia was associated with decreased levels of phosphatidylcholines and acylcarnitines and increased levels of polyunsaturated fatty acids, favouring an improvement of the atherogenic lipid profile [39]. Thus, we would expect a favourable effect of statins towards a normalisation of the plasma lipidome which could rather attenuate the associations between HRV indices and lipid metabolites observed herein.

Although dyslipidaemia is increasingly recognised as an important factor contributing to the pathogenesis of neuropathy, particularly in type 2 diabetes [3, 4], it is currently not well understood whether specific lipid classes and levels in the nerve are impacted [40]. It cannot be determined from this study whether the observed increase in systemic lipid levels in relation to lower HRV mirrors the local content in the peripheral nerves in diabetes. However, there is emerging evidence suggesting that the nerve concentrations of lipid metabolites such as phosphatidylcholines and SMs are elevated in mouse models of type 2 diabetes [41]. Recently, a dysregulation of lipid pathways with an increase in triacylglycerols containing saturated fatty acids and increased expression of diacylglycerol acyltransferase 2 (DGAT2), the enzyme required for the last step in triacylglycerol synthesis, was identified. Increased DGAT2 expression was present not only in nerves assessed in murine models of type 2 diabetes but also in sural nerve biopsies from hyperlipidaemic individuals with diabetes and peripheral neuropathy. These findings support the hypothesis that abnormal nerve–lipid signalling is an important factor in the pathogenesis of neuropathy in type 2 diabetes [40]. However, to date, no such experimental and clinical evidence is available for autonomic nerves.

This study has several strengths. First, it has a relatively large sample size of individuals with well-controlled type 1 and type 2 diabetes who underwent comprehensive metabolic characterisation assessed by state-of-the-art methodology. Second, HRV was determined during a 3 h hyperinsulinaemic–euglycaemic clamp, avoiding the impact of confounding factors such as blood glucose fluctuations [42]. Third, rigorous adjustment for numerous possible confounders and for multiple testing was applied. Yet this study also has some limitations. First, the cross-sectional design for the lipid metabolites and the relatively short follow-up period without deterioration in HRV after 5 years do not provide insight into the temporal sequence of the observed associations. Second, although a control group with normal glucose tolerance and HRV measurements is part of the GDS, lipidomics data for comparison with the diabetes groups were not available. Thus, it cannot be unequivocally determined from this study whether the observed associations of higher plasma levels of specific phosphatidylcholines and SMs with lower HRV indices can be attributed specifically to type 2 diabetes. However, indirect evidence suggests that such a scenario is conceivable, since on the one hand no such associations were found in recent-onset type 1 diabetes and on the other hand the systemic concentrations of both phosphatidylcholines and SMs predict the development of type 2 diabetes [11, 26, 27]. Third, statistical power of the prospective analysis was limited due to the relatively high number of dropouts at 5 years.

In conclusion, using targeted lipidomics we demonstrated that higher plasma levels of phosphatidylcholines and SMs are closely associated with lower cardiac vagal tone in individuals recently diagnosed with type 2 diabetes as opposed to those with type 1 diabetes. However, since cardiac autonomic function did not deteriorate over 5 years, analysis of the predictive value of lipid metabolites for the progression to CAN was not readily feasible. Further follow-up of the GDS participants over 10 and more years will presumably reveal deterioration in HRV in both participants with type 1 diabetes and type 2 diabetes, a prerequisite to allow for a more promising prediction analysis. Thus, plasma lipid panels could not only be useful to improve the prediction of the longer-term development or progression of CAN but also may allow for the clinical stratification of patients early in the course of the disease to target interventions in a more individualised approach to particularly susceptible patients. It has been suggested that given the key role of lipids in the pathophysiology of type 2 diabetes and CVD, lipidomics in general has the potential to improve prediction of future disease risk, inform on mechanisms of disease pathogenesis, identify patient groups responsive to particular therapies, and more closely monitor response to therapy. The ultimate utility of lipidomics to clinical practice will depend on: (1) its ability to predict future risk of morbidity and mortality when incorporated into conventional clinical risk engines; and (2), for widespread application, lipidomic-based measurements must be practical and accessible through standard pathology laboratories [43]. It remains to be established whether targeted lipidomics could be helpful in developing novel, potentially disease-modifying lipid-lowering treatment modalities.

## Electronic supplementary material

ESM (PDF 177 kb)

## Data Availability

The data that support the findings of this study are available from the GDS but restrictions apply to the availability of these data, which were used under license for the current study and therefore are not publicly available. Data are however available from the authors upon reasonable request and with permission of the GDS.

## Abbreviations

CAN: Cardiac autonomic neuropathy
DGAT2: Diacylglycerol acyltransferase 2
GDS: German Diabetes Study
HF: High-frequency
HPFS: Health Professionals Follow-up Study
HRV: Heart rate variability
LOD: Limit of detection
LF: Low-frequency
NHS: Nurses’ Health Study
NN: Normal-to-normal
PCaa: Diacyl-phosphatidylcholine
PCae: Acyl-alkyl- phosphatidylcholine
pNN50: Number of pairs of adjacent normal-to-normal intervals differing by >50 ms in the entire recording divided by the total number of normal-to-normal intervals
RMSSD: Root mean square of successive differences
SDNN: SD of all normal-to-normal intervals
SM: Sphingomyelin
TMAO: Trimethylamine-*N*-oxide
VLF: Very-low-frequency
