# Supplementary material for: Association of cardiac autonomic dysfunction with higher levels of plasma lipid metabolites in recent-onset type 2 diabetes
Source: Diabetologia. 2020 Oct 21;64(2):458–68. doi: 10.1007/s00125-020-05310-5 (PMC7801358; doi:10.1007/s00125-020-05310-5)
Supplement: Supplementary file 1 — (PDF 177 kb) [file 125_2020_5310_MOESM1_ESM.pdf]

Electronic Supplementary Material [ESM]

Association of cardiac autonomic dysfunction with higher levels of plasma lipid metabolites in recent-onset type 2 diabetes

Dan Ziegler[1,2,3], Alexander Strom[1,3], Klaus Straßburger[2,3], Birgit Knebel[3,5], Gidon J. Bönhof[1,2], Jörg Kotzka[3,5], Julia Szendroedi[1,2,3], Michael Roden[1,2,3], for the GDS group

[1]Institute for Clinical Diabetology, German Diabetes Center (DDZ), Leibniz Center for Diabetes Research at Heinrich Heine University, Düsseldorf, Germany;

[2]Division of Endocrinology and Diabetology, Medical Faculty, Heinrich Heine University, Düsseldorf, Germany

[3]German Center for Diabetes Research (DZD), München-Neuherberg, Germany;

[4]Institute for Biometrics and Epidemiology, German Diabetes Center (DDZ), Leibniz Center for Diabetes Research at Heinrich Heine University, Düsseldorf, Germany;

[5]Institute for Clinical Biochemistry and Pathobiochemistry, German Diabetes Center (DDZ), Leibniz Center for Diabetes Research at Heinrich Heine University, Düsseldorf, Germany.

ESM Methods

Heart rate variability indices

To briefly explain the physiologic basis of the various HRV indices, the simplest variable to calculate HRV is the SDNN, i.e. the square root of variance. Since variance is mathematically equal to total power of spectral analysis, SDNN reflects all the cyclic components responsible for variability in the period of recording reflecting both vagal and sympathetic influences on HRV. Measures derived from interval differences such as RMSSD, SD, and pNN50 represent measures of short-term variation reflecting high frequency variations in heart rate and thus are highly correlated. Among the frequency domain HRV parameters, the HF power reflects respiratory influences on the heart rate or respiratory sinus arrhythmia. The representation of LF and HF in normalized units emphasizes the controlled and balanced behavior of the sympathetic and parasympathetic branches of the autonomic nervous system, respectively (1). The physiological explanation of the VLF band is much less defined, but it depends primarily on the presence of parasympathetic outflow and may relate to the renin-angiotensin-aldeosterone system, thermoregulation, and/or peripheral vasomotor tone (2).

References

1. Task Force of the European Society of Cardiology and the North American Society of Pacing and Electrophysiology. Heart rate variability: standards of measurement, physiological interpretation and clinical use. Circulation 1996; 93:1043-1065.
2. Draghici AE, Taylor JA. The physiological basis and measurement of heart rate variability in humans. J Physiol Anthropol 2016; Sep 28;35:22.

ESM Table 1 – Relationship between NEFA and HRV measures in participants with recent-onset type 2 diabetes (*P*>0.05 after Bonferroni correction except for the underlined values).

	**Time domain HRV indices**								**Frequency domain HRV indices**					
	**pNN50**		**RMSSD**		**SDNN**		**SD**		**VLF**		**LF**		**HF**	
**FFA**	*r*	*P*	*r*	*P*	*r*	*P*	*r*	*P*	*r*	*P*	*r*	*P*	*r*	*P*
C14:0	-0.226	0.001	-0.200	0.004	-0.262	0.0002	-0.228	0.001	-0.233	0.001	-0.202	0.005	-0.183	0.011
cC14:1w5	-0.192	0.006	-0.170	0.015	-0.253	0.0002	-0.224	0.001	-0.253	0.0003	-0.186	0.009	-0.127	0.073
C16:0	-0.227	0.001	-0.200	0.004	-0.278	<0.0001	-0.253	0.0003	-0.243	0.001	-0.232	0.001	-0.203	0.004
cC16:1w7	-0.185	0.008	-0.159	0.022	-0.271	<0.0001	-0.241	0.0005	-0.258	0.0002	-0.195	0.006	-0.133	0.061
C18:0	-0.206	0.003	-0.192	0.006	-0.213	0.002	-0.199	0.004	-0.185	0.009	-0.167	0.019	-0.182	0.010
cC18:1w9	-0.198	0.005	-0.166	0.017	-0.215	0.002	-0.202	0.004	-0.201	0.004	-0.140	0.049	-0.152	0.032
cC18:2w6	-0.210	0.003	-0.191	0.006	-0.258	0.0002	-0.241	0.0005	-0.233	0.001	-0.226	0.001	-0.200	0.005
cC20:4w6	-0.162	0.021	-0.163	0.020	-0.135	0.054	-0.122	0.081	-0.080	0.260	-0.149	0.036	-0.173	0.015
cC22:4w6	-0.139	0.047	-0.134	0.055	-0.166	0.017	-0.158	0.023	-0.172	0.015	-0.113	0.114	-0.115	0.107
cC22:5w3DPA	-0.171	0.015	-0.142	0.041	-0.220	0.001	-0.198	0.004	-0.217	0.002	-0.214	0.002	-0.167	0.019

r and *P*-value for Spearman rank correlation; no associations were observed after adjustment for sex, age, BMI, smoking status, HbA1c, fasting blood glucose, M-value, triglycerides, cholesterol, HDL, LDL, creatinine, proteinuria, insulin therapy, oral antidiabetic drugs, antihypertensive drugs, and lipid lowering drugs.

ESM Table 2 – Relationship between phosphatidylcholines (PC) and lysophosphatidylcholines (lysoPC) and HRV measures in participants with recent-onset type 2 diabetes (*P*>0.05 after Bonferroni correction).

	**Time domain HRV indices**								**Frequency domain HRV indices**					
	**pNN50**		**RMSSD**		**SDNN**		**SD**		**VLF**		**LF**		**HF**	
**PCaa**	*r*	*P*	*r*	*P*	*r*	*P*	*r*	*P*	*r*	*P*	*r*	*P*	*r*	*P*
C32:2	-0.144	0.039	-0.142	0.042	-0.231	0.001	-0.213	0.002	**-0.236**	**0.001**	-0.190	0.007	-0.103	0.149
C34:3	-0.138	0.048	-0.110	0.115	**-0.233**	**0.001**	-0.225	0.001	**-0.236**	**0.001**	-0.173	0.014	-0.069	0.335
C34:4	-0.079	0.261	-0.095	0.173	-0.168	0.016	-0.146	0.036	-0.136	0.047	-0.131	0.065	-0.060	0.397
C36:1	-0.193	0.006	-0.177	0.011	-0.218	0.002	-0.222	0.001	-0.200	0.005	-0.156	0.028	-0.121	0.089
C36:2	-0.165	0.018	-0.147	0.035	-0.201	0.004	-0.207	0.003	-0.213	0.003	-0.174	0.014	-0.130	0.068
C36:3	-0.122	0.081	-0.106	0.131	-0.206	0.003	-0.210	0.002	**-0.225**	**0.001**	-0.143	0.045	-0.072	0.309
C36:4	-0.083	0.234	-0.112	0.109	-0.157	0.024	-0.155	0.026	-0.160	0.024	-0.122	0.085	-0.101	0.154
C40:4	-0.080	0.254	-0.081	0.249	-0.150	0.031	-0.158	0.024	-0.165	0.020	-0.113	0.110	-0.049	0.494
C42:2	-0.132	0.059	-0.122	0.082	-0.188	0.007	-0.178	0.010	-0.127	0.075	**-0.203**	**0.004**	-0.142	0.045
**PCae**<br>C30:0	-0.139	0.046	-0.127	0.070	-0.195	0.005	-0.171	0.014	**-0.214**	**0.002**	-0.174	0.014	-0.117	0.100
C32:1	-0.164	0.019	**-0.145**	**0.038**	**-0.223**	**0.001**	**-0.217**	**0.002**	**-0.218**	**0.002**	**-0.215**	**0.002**	**-0.137**	**0.053**
C34:0	-0.181	0.009	-0.184	0.008	-0.251	0.0003	-0.221	0.001	-0.251	0.0003	-0.240	0.001	-0.179	0.011
C34:1	-0.173	0.013	-0.149	0.033	-0.232	0.001	-0.211	0.002	**-0.243**	**0.001**	-0.202	0.004	-0.128	0.072
**lysoPC**<br>C16:0	-0.106	0.131	-0.099	0.159	**-0.169**	**0.015**	**-0.152**	**0.030**	**-0.208**	**0.003**	**-0.203**	**0.004**	**-0.097**	**0.173**
C16:1	**-0.131**	**0.062**	**-0.130**	**0.062**	**-0.201**	**0.004**	**-0.178**	**0.010**	**-0.201**	**0.004**	**-0.181**	**0.011**	**-0.104**	**0.145**

r and *P*-value for Spearman rank correlation (all *P*-values are not significant after Bonferroni correction); **boldface** indicates $P < 0.05$ after adjustment for sex, age, BMI, smoking status, HbA1c, fasting blood glucose, M-value, triglycerides, cholesterol, HDL, LDL, creatinine, proteinuria, insulin therapy, oral antidiabetic drugs, antihypertensive drugs, and lipid lowering drugs.
Abbreviations: aa, diacyl; ae, acyl-alkyl; lysoPC, lysophosphatidylcholines

ESM Table 3 – Relationship between sphingomyelins (SM) and HRV measures in participants with recent-onset type 2 diabetes (P>0.05 after Bonferroni correction).

	**Time domain HRV indices**								**Frequency domain HRV indices**					
	**pNN50**		**RMSSD**		**SDNN**		**SD**		**VLF**		**LF**		**HF**	
**SM**	*r*	*P*	*r*	*P*	*r*	*P*	*r*	*P*	*r*	*P*	*r*	*P*	*r*	*P*
C18:0	-0.180	0.010	-0.179	0.010	-0.220	0.001	-0.209	0.003	-0.190	0.007	-0.165	0.020	-0.147	0.039
C24:0	-0.178	0.011	-0.177	0.011	**-0.191**	**0.006**	**-0.192**	**0.006**	**-0.143**	**0.045**	**-0.125**	**0.079**	-0.165	0.020
C24:1	**-0.177**	**0.011**	**-0.151**	**0.030**	**-0.193**	**0.005**	**-0.184**	**0.008**	**-0.142**	**0.045**	**-0.176**	**0.013**	**-0.163**	**0.02**

r and *P*-value for Spearman rank correlation (all *P*-values are not significant after Bonferroni correction); **boldface** indicates $P < 0.05$ after adjustment for sex, age, BMI, smoking status, HbA1c, fasting blood glucose, M-value, triglycerides, cholesterol, HDL, LDL, creatinine, proteinuria, insulin therapy, oral antidiabetic drugs, antihypertensive drugs, and lipid lowering drugs.

ESM Table 4 – Relationship between acylcarnitines (AC) and HRV measures in participants with recent-onset type 2 diabetes (P>0.05 after Bonferroni correction).

	Time domain HRV indices								Frequency domain HRV indices					
	pNN50		RMSSD		SDNN		SD		VLF		LF		HF	
**AC**	*r*	*P*	*r*	*P*	*r*	*P*	*r*	*P*	*r*	*P*	*r*	*P*	*r*	*P*
C14:1	-0.179	0.010	-0.154	0.027	-0.222	0.001	-0.211	0.002	**-0.218**	**0.002**	-0.191	0.007	-0.202	0.004
C16	-0.210	0.002	-0.192	0.006	-0.229	0.001	-0.212	0.002	-0.230	0.001	-0.213	0.003	-0.232	0.001
C18	-0.181	0.010	-0.177	0.011	-0.144	0.039	-0.133	0.057	-0.118	0.096	-0.142	0.046	-0.204	0.004
C18:1	-0.157	0.025	-0.139	0.046	-0.164	0.019	-0.162	0.020	-0.160	0.024	-0.138	0.051	-0.170	0.016

r and *P*-value for Spearman rank correlation (all *P*-values are not significant after Bonferroni correction); **boldface** indicates $P < 0.05$ after adjustment for sex, age, BMI, smoking status, HbA1c, fasting blood glucose, M-value, triglycerides, cholesterol, HDL, LDL, creatinine, proteinuria, insulin therapy, oral antidiabetic drugs, antihypertensive drugs, and lipid lowering drugs.

ESM Table 5 – Relationship between lipid metabolites and HRV measures in participants with recent-onset type 1 diabetes (listed are only bivariate correlations with P<0.005 for at least one HRV index before adjustment; P>0.05 for all correlations after adjustment for sex, age, BMI, smoking status, HbA1c, fasting blood glucose, M-value, triglycerides, cholesterol, HDL, LDL, creatinine, proteinuria, insulin therapy, oral antidiabetic drugs, antihypertensive drugs, and lipid lowering drugs).

	Time domain HRV indices								Frequency domain HRV indices					
	pNN50		RMSSD		SDNN		SD		VLF		LF		HF	
**Metabolite**	***r***	***P***	***r***	***P***	***r***	***P***	***r***	***P***	***r***	***P***	***r***	***P***	***r***	***P***
AC C3	-0.267	0.007	-0.275	0.006	-0.165	0.101	-0.161	0.110	-0.098	0.347	-0.131	0.207	-0.294	0.004
PCaa C40:5	-0.273	0.006	-0.275	0.006	-0.294	0.003	-0.307	0.002	-0.189	0.054	-0.201	0.051	-0.238	0.020
PCaa C40:6	-0.325	0.001	-0.318	0.001	-0.330	0.001	-0.310	0.002	-0.217	0.035	-0.299	0.003	-0.335	0.001
PCaa C42:5	-0.229	0.023	-0.220	0.028	-0.280	0.005	-0.269	0.007	-0.223	0.030	-0.217	0.036	-0.232	0.036
lysoPC aC20:3	0.150	0.137	0.143	0.155	0.248	0.013	0.214	0.032	0.307	0.003	0.382	0.0001	0.151	0.143
lysoPC aC20:4	0.124	0.217	0.117	0.245	0.227	0.023	0.205	0.041	0.292	0.004	0.318	0.002	0.147	0.155
SM OH C22:2	-0.234	0.019	-0.234	0.019	-0.309	0.002	-0.265	0.008	-0.208	0.043	-0.270	0.008	-0.205	0.047
SM C16:1	-0.232	0.020	-0.234	0.019	-0.299	0.003	-0.275	0.006	-0.263	0.010	-0.228	0.026	-0.230	0.025
SM C18:0	-0.291	0.003	-0.282	0.004	-0.262	0.009	-0.254	0.011	-0.209	0.042	-0.163	0.114	-0.306	0.003
SM C18:1	-0.265	0.008	-0.268	0.007	-0.315	0.001	-0.313	0.002	-0.272	0.008	-0.218	0.034	-0.253	0.013

Abbreviations: aa, diacyl; PC, phosphatidylcholines; lysoPC, lysophosphatidylcholines; AC, acylcarnitines; SM, sphingomyelins.

ESM Table 6 – Demographic and clinical characteristics at baseline and 5-years follow-up

Variable	Type 1 diabetes (n=60)		Type 2 diabetes (n=95)	
	**Baseline**	**5-years**	**Baseline**	**5-years**
n (% male)	60 (68)		95 (71)	
Age (years)	34.9±13.2	40.0±13.1*	53.6±10.5	58.7±10.5*
BMI (kg/m²)	24.1±3.8	25.5±3.2*	32.0±5.9	31.9±6.0
Current smoking status (% yes)	18.3	20.0	16.8	16.8
Heart rate (bpm)§	68.9±10.1	70.8±10.0	70.1±9.3	71.2±9.7
Systolic blood pressure (mmHg)§	118±13	121±13	131±14	132±16
Diastolic blood pressure (mmHg)§	66.1±9.1	67.2±8.1	73.5±8.6	72.2±8.7
Triglycerides (mmol/l)§	0.72 (0.54, 0.97)	0.81 (0.60, 1.16)*	1.57 (1.12, 2.32)	1.84 (1.31, 2.74)*
Cholesterol (mmol/l)§	4.58±0.96	4.84±1.03*	5.39±1.01	5.48±1.07
HDL cholesterol (mmol/l)§	1.55±0.42	1.71±0.54*	1.19±0.33	1.27±0.38*
LDL cholesterol (mmol/l)§	2.63±0.82	2.81±0.84*	3.36±0.90	3.55±0.92*
Creatinine (nmol/l)	78.5±12.8	76.0±13.4	82.1±15.0	77.4±16.4*
HbA1c (%)<br>HbA1c (mol/mmol)	6.6±1.0<br>48.6±11.1	6.9±1.0*<br>52.2±10.5*	6.3±0.7<br>45.6±7.9	6.8±0.9*<br>50.3±9.9*
Fasting glucose (mmol/l)	7.78±2.74	8.21±2.99	7.05±1.68	8.18±2.15*
M-value (mg * kg^{-1} * min^{-1})§	8.32 (5.98, 10.40)	6.38 (4.72, 8.10)*	6.22 (4.27, 7.81)	5.79 (4.53, 7.29)
Antihypertensive drugs (%)	3.8	13.2	65.4	80.2*
Albuminuria (mg/l)§	14.9±11.2	7.2±21.2*	17.5±17.2	14.1±51.6*
Subclinical/borderline CAN (%)	2.1	0.0	4.5	7.6
Definite CAN (%)	0.0	4.2	3.0	10.6

Data are shown as %, mean±SD, or median (first, third quartile); *p<0.05 vs baseline.

Abbreviations: CAN, cardiovascular autonomic neuropathy; HDL, high-density lipoprotein; LDL, low-density lipoprotein.

ESM Table 7 – Follow-up of heart rate variability (HRV) after 5 years.

	**Type 1 diabetes**		**Type 2 diabetes**	
**HRV variables**	**Baseline**	**5 years[a]**	**Baseline**	**5 years[a]**
Time domain HRV indices pNN50 (%)	12.6 (6.4, 25.5)	12.1 (5.2, 30.0)	5.3 (2.0, 18.3)	5.5 (2.4, 14.4)
RMSSD (ms)	37.8 (28.7, 52.0)	34.9 (26.9, 56.6)	28.8 (21.4, 43.4)	29.7 (23.4, 43.4)
SDNN (ms)	66.7 (52.5, 86.7)	71.6 (51.7, 90.8)	51.9 (41.1, 67.5)	53.2 (42.7, 71.2)
SD (ms)	72.8 (58.9, 90.5)	**83.3 (67.2, 100.2)**	62.8 (48.7, 79.9)	**70.7 (56.6, 87.9)**
Frequency domain HRV indices VLF power (ms^2)	2015 (1437, 3263)	**2767 (1718, 3930)**	1466 (871, 2601)	1647 (1034, 2676)
LF power (ms^2)	1209 (748, 2143)	1373 (706, 2094)	659 (391, 1170)	614 (339, 1053)
HF power (ms^2)	351 (188, 743)	342 (203, 809)	170 (98, 354)	189 (107, 396)

Data are shown as median (first, third quartile). Boldface indicates $p<0.05$ vs baseline (Wilcoxon signed-rank test). [a]Values represent the sum of the original values and the added age-dependent 5-year changes in the corresponding HRV indices obtained from the regressions computed in glucose-tolerant individuals from the GDS study.

Following equations/syntaxes for 5-years follow-up age adjustment, calculated using 167 glucose-tolerant individuals from the GDS study, were applied:

```
COMPUTE pNN50_change=EXP(4.676 - 0.063 * AGE_follow-up) - EXP(4.676 - 0.063 * AGE_baseline). EXECUTE.
COMPUTE RMSSD_change=EXP(4.490 - 0.022 * AGE_follow-up) - EXP(4.490 - 0.022 * AGE_baseline). EXECUTE.
COMPUTE SDNN_change=EXP(4.764 - 0.014 * AGE_follow-up) - EXP(4.764 - 0.014 * AGE_baseline). EXECUTE.
COMPUTE SD_change=EXP(4.988 - 0.013 * AGE_follow-up) - EXP(4.988 - 0.013 * AGE_baseline). EXECUTE.
COMPUTE VLF_change=EXP(8.810 - 0.025 * AGE_follow-up) - EXP(8.810 - 0.025 * AGE_baseline). EXECUTE.
COMPUTE LF_change=EXP(8.226 - 0.034 * AGE_follow-up) - EXP(8.226 - 0.034 * AGE_baseline). EXECUTE.
COMPUTE HF_change=EXP(7.727 - 0.050 * AGE_follow-up) - EXP(7.727 - 0.050 * AGE_baseline). EXECUTE.
```
